# Biomarkers of non-communicable chronic disease: an update on contemporary methods

**DOI:** 10.7717/peerj.12977

**Published:** 2022-02-24

**Authors:** Solaiman M. Al-hadlaq, Hanan A. Balto, Wail M. Hassan, Najat A. Marraiki, Afaf K. El-Ansary

**Affiliations:** 1 Department of Restorative Dental Sciences, College of Dentistry, King Saud University, Riyadh, Saudi Arabia; 2 Central Research Laboratory, Female Campus, King Saud University, Riyadh, Saudi Arabia; 3 Department of Biomedical Sciences, University of Missouri-Kansas City School of Medicine, Kansas City, KS, United States of America; 4 Department of Botany and Microbiology, College of Science, King Saud University, Riyadh, Saudi Arabia

**Keywords:** Biomarkers, Chronic diseases, Multivariate analysis, Inflammation, Oxidative stress, Mitochondrial dysfunction, Principal component analysis

## Abstract

Chronic diseases constitute a major global burden with significant impact on health systems, economies, and quality of life. Chronic diseases include a broad range of diseases that can be communicable or non-communicable. Chronic diseases are often associated with modifications of normal physiological levels of various analytes that are routinely measured in serum and other body fluids, as well as pathological findings, such as chronic inflammation, oxidative stress, and mitochondrial dysfunction. Identification of at-risk populations, early diagnosis, and prediction of prognosis play a major role in preventing or reducing the burden of chronic diseases. Biomarkers are tools that are used by health professionals to aid in the identification and management of chronic diseases. Biomarkers can be diagnostic, predictive, or prognostic. Several individual or grouped biomarkers have been used successfully in the diagnosis and prediction of certain chronic diseases, however, it is generally accepted that a more sophisticated approach to link and interpret various biomarkers involved in chronic disease is necessary to improve our current procedures. In order to ensure a comprehensive and unbiased coverage of the literature, first a primary frame of the manuscript (title, headings and subheadings) was drafted by the authors working on this paper. Second, based on the components drafted in the preliminary skeleton a comprehensive search of the literature was performed using the PubMed and Google Scholar search engines. Multiple keywords related to the topic were used. Out of screened papers, only 190 papers, which are the most relevant, and recent articles were selected to cover the topic in relation to etiological mechanisms of different chronic diseases, the most recently used biomarkers of chronic diseases and finally the advances in the applications of multivariate biomarkers of chronic diseases as statistical and clinically applied tool for the early diagnosis of chronic diseases was discussed. Recently, multivariate biomarkers analysis approach has been employed with promising prospect. A brief discussion of the multivariate approach for the early diagnosis of the most common chronic diseases was highlighted in this review. The use of diagnostic algorithms might show the way for novel criteria and enhanced diagnostic effectiveness inpatients with one or numerous non-communicable chronic diseases. The search for new relevant biomarkers for the better diagnosis of patients with non-communicable chronic diseases according to the risk of progression, sickness, and fatality is ongoing. It is important to determine whether the newly identified biomarkers are purely associations or real biomarkers of underlying pathophysiological processes. Use of multivariate analysis could be of great importance in this regard.

## Introduction

A biomarker is a tool that is used and employed by doctors, health professionals, and scientists to measure and indicate a pathogenic process, biological process, or an exposure or intervention response (Institute of Medicine US Committee on Qualification of Biomarkers and Surrogate Endpoints in Chronic Disease, 2010; Yetley, De Mets & Harlan Jr, 2017). It is essential to outline the analytical features that makes a biomarker useful in reflecting the course and activity of any disease. Ideally, a biomarker should be readily available, reproducible, and measurable in biological samples such as blood, urine, cerebrospinal fluid, and saliva (Lee et al., 2013). It should also be non-invasive, inexpensive, and have a high specificity and sensitivity to allow early detection. The advantage of this clinical feature allows for accurate differentiation between the normal and pathological status, as well as other clinical conditions (Devarajan, 2007). Detailed understanding of disease biology can potentially increase the effectiveness of prevention and treatment approaches, in addition to decreasing adverse effects from helpful treatments. Progress of biomarkers is critical in these areas, and it is vital that adequate studies of newly discovered biomarkers are performed before adopting their use in routine clinical management.

Biomarkers can be simply classified into, diagnostic, predictive, and prognostic markers. A diagnostic biomarker refers to a biological variable that helps the diagnosis of a disease and may aid in determining disease progression and/or success of treatment. It could be a laboratory, radiological, anatomical, physiological or other measurable parameter that helps to differentiate one disease from others.

A predictive biomarker is utilized to designate individuals that have a greater possibility of responding to exposure to a particular environmental agent or medical product, compared to their condition at baseline (Verdaguer, Saurí & Macarulla, 2017). This response can vary from being an adverse effect, a symptomatic advantage, or improved survival. An example of where a predictive biomarker is used is during a randomized controlled clinical trial of an investigational therapy, to enrich the study population for a medical product development.

A prognostic biomarker is employed to provide information on the possible health outcome of a patient irrespective of the treatment (Sechidis et al., 2018). It is used to indicate the possibility of disease recurrence, future clinical events, or disease progression in an identified population, and is usually measured at a defined baseline. It is common, in the biomedical field, to apply the term prognostic in healthy individuals who have a likelihood of developing a disease or a certain diagnosis in the future in addition to individuals who have been diagnosed with a certain disease or medical condition (Califf, 2018).

Chronic disease is defined as an illness of long duration, commonly slow in progression (WHO, 2021c). In 2013, the Global Burden of Disease study reported a significant increase in the years lived with disability (YLD) among patients with chronic diseases (Global Burden of Disease Study 2013 Collaborators, 2015). Multi-morbidity associated with chronic conditions is notably high in developed countries (with significant increase in its prevalence with the increase in age (Dennis, 2016). In fact, multi-morbidity is reported to be around 50% for 65–74-year-olds, increasing to 70% for 85 years old or over (Chronic disease Overview, 2021). Addressing chronic disease is a major challenge for healthcare systems around the world, which have been largely developed to deal with acute episodic care, rather than to provide a systematic approach to provide an organized care for people with long-term conditions (WHO, 2021b), which are frequently characterized by the need for long periods of supervision, observation or care.

Patients with chronic diseases usually face multiple challenges. The high cost of healthcare frequently needed by chronic disease patients is one of the top challenges. In addition, Patients with chronic disease usually experience major or minor physical disability concomitant with their illness which compromises their ability to sustain their lifestyle. Furthermore, emotional complications such as fear, anger, and depression are also of great importance as patients realize that it may be impossible to be cured. Additional care for patients with more than one chronic condition usually constitutes added burden requiring additional medical care and expenses compared to the average patient (Allen et al., 2014).

Chronic disease is a broad category that encompasses non-communicable diseases (NCDs), such as diabetes, osteoarthritis, heart disease, chronic obstructive pulmonary disease, cancer, and depression, as well as communicable diseases (CDs), such as acquired immunodeficiency syndrome (AIDS) and hepatitis. In the biomedical field, the diagnosis of (NCDs) can be categorized according to etiology, pathophysiology, protracted clinical course, comorbidity, symptoms, complications and treatment. However, they all involve an expected long duration and absence of a definitive cure (Bernell & Howard, 2016).

**Figure 1:**
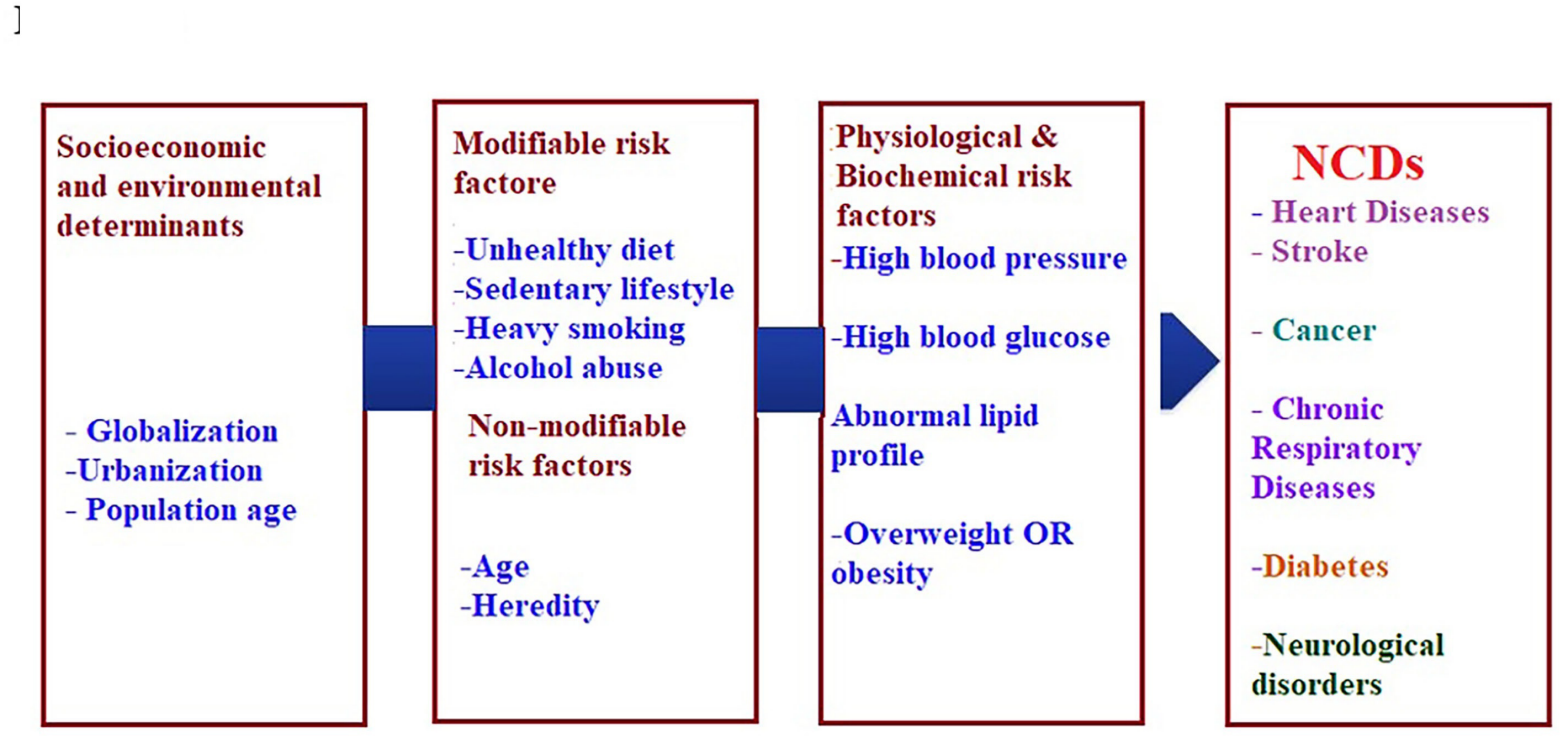
Risk factors of non-communicable chronic diseases.

The current burden of NCDs is attributed to the earlier exposure to accumulative health risks, while the future burden can be related to the current exposure of population to multiple risk factors that can be either non-modifiable such as age, gender, and genetic vulnerability or modifiable such as diet, and physical activity (WHO, 2021a; Yach et al., 2004). Although the linkage between risk factors and chronic disease is comparable globally, it is greater in developing countries (Rodgers et al., 2004). Figure 1. Summarizes the modifiable and non-modifiable risk factors that are involved in the etiology of (NCDs).

Worldwide, it is generally believed that chronic diseases constitute the most common reason of death, it is led by heart disease (17 million deaths), followed by cancer (7 million deaths), chronic lung disease (4 million) and diabetes mellitus (1.6 million) (World Health Organization, 2005). Other fundamental factors are the key powers motivating economic, cultural, and community change, including globalization, urbanization, and the overall environmental influences (WHO, 2021d). In addition, global powers such as trade and marketing are enhancing the increase in the incidence of chronic diseases. For example, one of the health-related effects of globalization is the trend known as ‘nutrition transition’ in which a traditional diet rich in fruit and vegetables is replaced by a diet rich in calories derived from animal fats, and lower in complex carbohydrates (Popkin, 2002). Such diet, when combined with a low level of physical activity, alcohol consumption, and heavy smoking usually results in global expansion of chronic diseases (WHO, 2021d). The association between modifiable lifestyle risk factors (alcohol, body mass index, smoking, diet, physical inactivity) and the age to have the first chronic disease were recently reported by Ng et al. (2020).

Additionally, Oral hygiene is considered as a central measure of overall (Allen & Camisa, 2015). Nevertheless, the high incidence of dental problems (DPs), such as periodontitis, and tooth loss, persist as public global health problems. DPs have been found to be linked with various CDs, including diabetes (Preshaw & Bissett, 2019), metabolic syndrome (Lamster & Pagan, 2017), cardiovascular diseases (CVD) (Sanz et al., 2020), lung (Gomes-Filho et al., 2020) and kidney problems (Delbove et al., 2021) in the general population.

While etiopathologies of common CDs are greatly different, they are associated with common and disease-specific alterations in the composition and function of the gut microbiota (Fan & Pedersen, 2021). Interestingly, when considering the translational associations of gut microbiome examination, it became apparent that most CDs and drugs have impact on microbiome, metatranscriptome and metabolome profiles in addition to their ascertained effects on CDs biomarkers (Adeshirlarijaney et al., 2019; Fan & Pedersen, 2021).

Mitochondria are cellular organelles that convert energetic substrates such as lipids, glucose, and oxygen into energy. These organelles have a double membrane, circular DNA molecules, and the ability to interact with each other (Braschi & McBride, 2010). Constant mitochondrion-mitochondrion communication occurs *via* dynamic interactions of membrane fusion and fission. These interactions change mitochondrial morphology and consecutively modulate the organelles’ function (Westermann, 2010).

Mitochondrial dysfunction, characterized by impaired oxidative phosphorylation as the major role of the electron transport chain, and reduction in the mitochondrial capability to produce adenosine triphosphate (ATP), is a common character of all chronic diseases (Green, Galluzzi & Kroemer, 2011; Swerdlow, 2011).

Mitochondrial dysfunction, chronic inflammation, and oxidative stress are involved in most NCDs diseases such as cancer, atherosclerosis, Parkinson’s disease, Alzheimer’s disease, other neurodegenerative diseases, heart and lung disturbances, diabetes, obesity, and autoimmune diseases (Ij, 2017). In addition to the impairment of mitochondrial function, obesity and obesity-related complications are universally associated with chronic disease.

For example, in muscle tissues, mitochondrial dysfunction leads to the reduction of fatty acid oxidation and the inhibition of glucose transport, indicating that insulin-stimulated glucose transport is reduced as a hallmark of insulin resistance and type 2 diabetes. Mitochondrial ROS have also been correlated to the amplified activity of uncoupling proteins (UCP), which uncouples ATP synthesis from electron transport. UCP activity leads to heat generation without ATP production. Long-term reductions in ATP levels, affect multiple cellular signaling mechanisms associated with chronic disease (Brondani et al., 2012; Fedorenko, Lishko & Kirichok, 2012). Usually, NCDs, such as cancer, obesity, and diabetes are accompanied with high ROS levels which serve as markers for oxidative stress and elevated concentrations of free fatty acids (FFA) (Matsuda & Shimomura, 2013). Since ROS and FFA are important molecular signals that activate UCP2, it is essential to understand and clarify the relationship between UCP2 and chronic diseases. UCP is a family of mitochondrial anion proteins existing in the inner mitochondrial membrane. Since their discovery, they have attracted significant attention due to their contribution to the mitochondrial-mediated oxidative stress and impaired energy metabolism. Therefore, UCP2 is an important target to consider in the efforts attempting to control the prevalence of multiple chronic diseases (Sreedhar & Zhao, 2017).

It is generally accepted nowadays, that mitochondrial dysfunction is involved in the etiological mechanisms of several chronic diseases, including cardiac, liver and kidney disorders, and neurodegenerative diseases (*e.g.*, Parkinson’s disease and Alzheimer’s disease) (Geto et al., 2020).

The use of biomarkers governed by the superiority of data that supports their use and on the view of their application. Evaluation of the quality of the laboratory assays and data associating the biomarkers to clinical outcomes is important for evaluating biomarker utility.

This encouraged us to survey the possibility of using multivariate statistical analysis in the field of biomarkers of chronic diseases. This could help to search for a discriminant, non-invasive, and easily applicable diagnostic tool. This might be of great help to care practitioners and their patients make decisions related to patient precaution.

## Survey methodology

In order to ensure a comprehensive and unbiased coverage of the literature, first a preliminary skeleton of the manuscript (title, headings and subheadings related to biomarkers and chronic diseases) was drafted by the authors working on this paper.

Second, based on the components drafted in the preliminary skeleton a comprehensive search of the literature was performed using the following search engines:

– PubMed
– Google Scholar

The used key words included:

– **Biomarker**
– **Chronic disease** (Diabetes mellitus, Cardiovascular diseases, Chronic kidney diseases, and neurological disorders)
– **Oxidative stress** (Role of Oxidative stress as etiological mechanisms of different chronic diseases)
– **Chronic inflammation** (As etiological mechanism in chronic diseases)
– **Quality of life of chronic disease patients** (Factors affecting QOL)
– **Oral disease** (Etiological mechanisms)
– **Dental Caries** (How it is related to chronic diseases)
– Oral Cancer
– Periodontal disease
– Multivariate (**Applications in Chronic Diseases)**

These keywords were used as single keywords and in various combinations. Efforts were made to focus on the most recent publications (2016 –present), however, older references were included as deemed necessary according to the requirements of proper coverage of the topic. Search results were scanned, and the most relevant papers were selected to ensure a comprehensive and balanced representation of the current schools of thoughts on the topic. The selected articles were further examined to identify more articles that could be used in the review.

### Chronic disease effect on patients’ quality of life

There are certain difficulties associated with assessing the multidimensional health of older patients with NCDs. Comprehensive assessment of the elderly patient’s health status requires a sophisticated set of measures that address their specific needs and cannot be merely copied from procedures designed for the general population. In fact, elderly patient’s quality of life evaluation (QOL) constitutes a significant factor that requires major attention. Currently, QOL is basically founded on subjective assessment with direct objective clinical criteria to evaluate patient’s QOL. Yang et al. (2012) found that lung damage leading to disturbed function was negatively correlated with the long-term QOL (Xie et al., 2005). Although, Pereira et al. (2020), was able to elucidate an impact of clinical objective parameters in diabetic patients on QOL scoring, it is yet to be formulated in a comprehensive manner to be useful in improving QOL in research on individuals diagnosed with diabetes indicated an impact of various clinical objective indicators on the scores of all fields of QOL indicators. Clarifying this correlation in the elderly can provide a scientific basis for improving QOL (emotional status or stress, bodily and societal ability, role performing, and clinically presentation or symptoms) (Ping et al., 2020).

To achieve a meaningful impact on the elderly QOL, the scientific community must overcome the shortcomings associated with the methods currently employed to achieve complete health evaluation of elderly patients with one or more chronic diseases. In fact, developing a simple, practical, and multidimensional system to evaluate the health of the elderly that is accurate and reproducible will impact their lives significantly. In effect, Forestier et al. (2019) proposed that investigators are required to develop precise experimental tools that can measure the capabilities of chronic patients. These tools should be multidimensional including measures of adaptation to illness, attitude, personal experience, and treatment or healthcare in relation to objective clinical indicators.

### Oral cavity problems and NCDs

The oral data taken out from the Global Burden of Disease Study in 2010 indicated that periodontal disease, oral cancer and caries increased noticeably by an average of 45.6% from 1,990 to 2010 together with the major NCDs like diabetes which raised by 69.0% (Murray & Lopez, 2013). Oral diseases are therefore recognized as a major global health burden, having huge impacts on people’s daily lives and economic development, with the loss of millions of school and work hours yearly around the world (Seitz et al., 2019). Therefore, the diagnosis of these active oral disease at an early stage would result in more predictable treatment outcomes and cost savings.

Saliva has been studied extensively as a potential diagnostic tool of multiple oral and systemic diseases due to its ease and non-invasive accessibility along with its abundance of biomarkers. In fact, almost 40% of proteins that may be biomarkers for cancer, cardiovascular disease and stroke can be found in whole saliva (Loo et al., 2010). Salivary analysis has great potential in large clinical trials, self-management of disease, epidemiologic studies in regulated clinical environments and in locations where access to clinical facilities is scarce (Jaedicke, Preshaw & Taylor, 2016).

Saliva can be a very effective tool for screening and detection of periodontal disease, observing treatment outcomes and identification of refractory or progressing cases. Detecting patients that might be at risk for future disease will help in improving risk management strategies, preventive care and/or behavior change on the part of the patient to prevent the onset of disease. In addition to saliva, gingival crevicular fluid (GCF) has been explored as a diagnostic and prognostic tool, which aims to demonstrate that the flow of gingival fluid is sufficiently indicative of the inflammatory state of periodontal tissues. To date, more than 90 different components in GCF have been evaluated for periodontal diagnosis (Loos & Tjoa, 2005). Collection and analysis of GCF has long been a popular approach to investigating localized inflammatory processes in periodontitis (Jaedicke, Preshaw & Taylor, 2016).

Head and neck cancer constitute a global health burden ranking as the seventh most prevalent cancer with about fifty percent of it arising in the oral cavity (Marur & Forastiere, 2008). Ninety percent of oral cancer is squamous cell carcinoma with a 5-year survival rate of about 50% (Curado & Boyle, 2013; Leoncini et al., 2015; Siegel, Miller & Jemal, 2019). This high mortality rate is attributed to the aggressive nature of the cancer combined with late diagnosis in stage III and IV (Bello, Soini & Salo, 2010; Kowalski & Carvalho, 2000). To improve diagnostic procedure as well as prediction of prognosis, biomarkers have been studied in the area of oral cancer (Almangush et al., 2018; Cristaldi et al., 2019). In fact, saliva has received significant attention as potential source of biomarkers for oral squamous cell carcinoma as well as other cancers (Aro, Kaczor-Urbanowicz & Carreras-Presas, 2019; Kaczor-Urbanowicz et al., 2017). Several diagnostic salivary biomarkers have been investigated including CD44 (Franzmann et al., 2005), Cyfra 21-1, tissue polypeptide antigen, and cancer antigen 125 (Nagler et al., 1999). It is evident that no single biomarker is sufficient to detect early stages of oral squamous cell carcinoma (Nagler et al., 1999), however, a panel of biomarkers have shown promising results for oral cancer detection (Li et al., 2004).

Periodontitis is characterized by inflammatory destruction of the alveolar bone as well as loss of the soft tissue attachment to the teeth. Periodontitis has three phases: inflammation; connective tissue degradation; and bone turnover. During each phase of the disease, specific host-derived biomarkers have been identified. However, it must be mentioned that an individual biomarker doesn’t have high sensitivity and specificity level to be considered as a diagnostic tool. Actually, panels of salivary biomarkers and recognized periodontal pathogens may offer promising applications for differential diagnosis, treatment planning and monitoring, as well as for identification of patients at risk for future tissue destruction (Miller et al., 2010; Zhang et al., 2010). Salivary matrix metalloproteinase-8, when used together with interleukin-1beta and *P. gingivalis* can be a more successful diagnostic tool to identify periodontitis than each marker alone (Gursoy et al., 2011; Salminen et al., 2014).

Dental caries development is associated with a number of factors, however, it has a high prevalence making it one of the most common chronic diseases (Petersen, 2003). It is caused by complex interactions among acid-producing bacteria, fermentable dietary carbohydrates, and several host factors including saliva. In fact, saliva is one of the most important host factors controlling the occurrence and progression of dental caries (Fejerskov, Nyvad & Kidd, 2009). Evidence concerning salivary proteins as biomarkers for dental caries were inconsistent and insufficient (Martins et al., 2013), however, studies suggested an association between dental caries and a number of salivary parameters, including abundance of *Streptococcus mutans* (Xiao et al., 2016), high pH, buffering capacity, and salivary flow rate (Bashir et al., 2019). Thick, sticky, and frothy saliva with an increased viscosity creates susceptible environment for caries (Kaur, Kwatra & Kamboj, 2012). Since dental caries is multifactorial disease, it is difficult to establish a single biochemical marker to predict the severity of the disease (Laputková et al., 2018). Microbial, immunological, oral hygiene, and sociodemographic variables remain important caries predictors and determine the occurrence and severity of clinical disease (Stojković et al., 2020).

Interestingly, Tu et al. (2018) reported a considerable effect of chronic disease on cancer risk, which was as important as lifestyle combined risk factors. More than one fifth of new cancer cases and over one third of cancer deceases were attributable to common chronic diseases such as cardiovascular disease, chronic renal disease, diabetes mellitus, gouty arthritis, and lung disease (Giovannucci et al., 2010; Grossman et al., 2002; Lowrance et al., 2014; Stocks et al., 2012). Physical activity is a promising approach to reduce chronic disease associated risk of cancer (Kruk & Czerniak, 2013). These findings have important implications for developing new cancer prevention strategies through improving the management of chronic diseases.

Public health authorities face extra challenges in avoiding communicable diseases, which are also called infectious or transmissible diseases. Communicable diseases are usually triggered by microorganisms such as bacteria, viruses, parasites and fungi that can spread, directly or indirectly, from one person to another. Some are transferred through bites from insects while others might develop through the ingestion of contaminated water or food. According to the WHO, the most prevalent communicable diseases are HIV/AIDS, tuberculosis, malaria and lower respiratory tract infections (Edemekong & Huang, 2021). Globally, death rates for community acquired pneumonia (CAP) extensively differ from country to country, fluctuating between <1% to 48% (Welte, Torres & Nathwani, 2012). A yearly prevalence of 150.7 million new cases is verified, of which 7–13% are severe enough that needs to be hospitalized (Regunath & Oba, 2021).

Although the current global prevalence of communicable diseases is declining due to the advancement in immunization programs and drug development; communicable diseases still constitute a significant threat. For example, increasing international travel raises the risk of transferring transmissible pathological microorganisms from one country to another. Fortunately, public health authorities are making great progress in rising the awareness of public health threats associated with infectious diseases (Arias-Fernández, Gil-Prieto & Gil-de-Miguel, 2020).

Established biomarkers of communicable chronic diseases consist of the inflammatory response components, hematological indicators of infection, and serology elements related to the infectious agents. Hematological factors, such as the white blood cell count (WBC), are generally considered an excellent marker of infection and inflammation. WBC or leukocytes are involved in the initial stages of the human body defenses against pathogenic invaders leading to increased WBC production, therefore, WBC count has often been used as an early infection biomarker (Smock & Perkins, 2013; Yusa et al., 2017). In addition, differential count of leukocytes has been used to help discriminate among various infectious disease conditions (31). Furthermore, erythrocyte sedimentation rate (ESR) and C-reactive protein (CRP) are considered conventional markers of infection and/or inflammation (Bray et al., 2016). Another set of well-known biomarkers are the serological markers which are used extensively in the diagnosis of infectious disease through direct identification of the causative agent or indicating the presence of an infection. Sero-diagnosis is based on “the principle that the reaction between an antibody and an antigen will result in a recordable event” with the aim of finding an antigen or an antibody to determine the microorganism associated with a particular condition (Ohst et al., 2018).

### General molecular mechanisms that drive chronic disease pathophysiology

#### Oxidative stress and chronic diseases

Aging is a common un-modifiable risk factor of most chronic diseases. It is a process that is characterized by the progressive dysfunction of tissues and organs. The theory of oxidative stress is based on the hypothesis that functional damage related to aging is a consequence of the buildup of destruction caused by reactive oxygen and nitrogen species (RONS).

Free radicals are highly reactive atoms or molecules with one or more unpaired electron(s) in their external shell (Lobo et al., 2010). These radicals can be generated in the cells by accepting or losing a single electron, consequently, behaving as reductants or oxidants (Lobo et al., 2010). Reactive oxygen species (ROS) and reactive nitrogen species (RNS) are two terms that refer to reactive radical and non-radical derivatives of oxygen and nitrogen, respectively (Powers, Talbert & Adhihetty, 2011). (Reactive oxygen and nitrogen species (RONS) are produced by all aerobic cells and play an important role in aging as well as in age-related diseases (Venkataraman, Khurana & Tai, 2013).

In diabetes mellitus, the exact mechanism by which oxidative stress may contribute to the progress of complications is not fully understood (Bashan et al., 2009). However, in type 2 diabetes (T2D), oxidative stress is believed to stimulate pro-thrombotic responses, leading to cardiovascular complications (De Cristofaro et al., 2003). Diabetes injuries can be measured as tissue-oxidative-damaging effects of chronic high blood glucose or hyperglycemia. Increased intracellular level of glucose leads to an extraordinary production of RONS, which surpasses the antioxidant ability of the cell to counteract them (Brownlee, 2001). In fact, RONS activate four important signaling pathways that contribute to the hyperglycemia-induced oxidative tissue damage. These four molecular pathways include activation of protein kinase C (PKC), stimulation of hexosamine pathway flux, increased advanced glycation end products (AGEs), and increased polyol pathway flux (Golbidi, Ebadi & Laher, 2011). It is noteworthy to mention that triggering the AGEs pathway can damage cells through the alteration of gene transcription of blood proteins such as albumin, initiating their binding to AGEs receptors (RAGEs) on macrophages and thus increase the production of growth factors and pro-inflammatory cytokines (Brownlee, 2005).

Cardiovascular disease (CVD) is considered a leading cause of death in the elderly. Atherosclerosis, which is closely related to CVD, is associated with oxidative stress as the etiopathological mechanism (Liguori et al., 2018). Numerous studies have confirmed that heart tolerance to oxidative stress declines with age because of a decrease in the level of some antioxidant enzymes such as glutathione peroxidase (GSH-Px) and superoxide dismutase (SOD), contributing to the development of CV changes (Abete et al., 1999).

Oxidative stress is believed to play an essential role in the progress of chronic kidney disease (CKD), through glomerular damage and kidney ischemia and, indirectly, through the effects of hypertension, inflammation, and endothelial dysfunction (Balasubramanian, 2013). CKD patients are in a chronic inflammation state characterized by the activation of inflammatory cells such as Polymorphonuclear leukocytes (PMNs) and monocytes. These inflammatory cells increase the secretion of nicotinamide adenine dinucleotide phosphate (NADPH) oxidase, myeloperoxidase (MPO), that enhance the production of ROS (Putri & Thaha, 2014). Moreover, leukocytes of CKD patients produce superoxide anions, which inactivate nitric oxide (NO), reducing the capacity of blood vessels dilatation that leads to hypertension.

Inflammation related oxidative stress is believed to play a considerable role in the pathogenesis of cancer. This association can be briefly explained as follows, RONS and inflammatory cytokines, such as TNF *α*, activate nuclear factor kappa-B (NFk-B) transcription factor which activates the expression of genes implicated in cell proliferation leading to uncontrolled cell division, and loss of programmed cell death or apoptosis of cancer cells (Zielinska et al., 2018). Chronic inflammation is also associated with angiogenesis, another characteristic of cancer. This is attributed to the fact that RONS can also increase the expression of certain transcriptional factors that are involved in neoplastic transformation and enhancement of cancer angiogenesis (Federico et al., 2007; Marnett, 2000). Among oxidized DNA radicals, 8-nitroguanine and 8-oxoguanine are greatly involved in the inflammation/oxidative stress-induced carcinogenesis especially in the elderly (Khansari, Shakiba & Mahmoudi, 2009).

In addition, oxidative stress mechanism has been shown to play a pivotal role in the pathophysiology of central nervous system leading to neurological disorders (NDs) such as Alzheimer’s disease (AD), Parkinson’s disease (PD), and Huntington’s disease (HD), as well as vascular dementia clinically presented as progressive loss of memory, impairments in the movement, or progressive inability to move (Abete et al., 2014; Cacciatore et al., 2005; Liu et al., 2017).

Interestingly, oxidative stress mechanisms have been implicated in the development and progression of periodontal disease (Sczepanik et al., 2020). Although microorganisms in dental plaque are the primary causative agent, it is believed that PMNs’ response to microbial plaque is associated with the overproduction of reactive oxygen species that lead to progressive soft tissue and bone destruction (Almerich-Silla et al., 2015; Baňasová et al., 2015; Chapple & Matthews, 2007; Konopka et al., 2007). It is noticeable that periodontal disease is associated with other illnesses that are linked with oxidative stress such as diabetes (Bullon, Newman & Battino, 2014).

#### Chronic inflammation and chronic diseases

Acute inflammatory response is described as a temporally short and controlled upregulation of inflammatory action that occurs as a response to a potential tissue damaging stimulus. It generally resolves as soon as the cause has receded (Fullerton & Gilroy, 2016; Kotas & Medzhitov, 2015; Straub, 2017). Nevertheless, the presence of certain psychological, environmental and/or biological risk factors has been implicated in the inhibition of the complete resolution of acute inflammation thereby, promoting a state of long-lived low-grade systemic chronic inflammation (SCI). It is characterized by the activation of immune mechanisms that are often different from those involved during the acute immune response (Calder et al., 2013; Miller & Raison, 2016). Shifts in the inflammatory response from short- to long-lived can cause a breakdown of immune tolerance leading to major changes in all tissues and organs, usually causing an increase in the risk for various NCDs in young and old individuals (Ferrucci & Fabbri, 2018; Kotas & Medzhitov, 2015; Miller & Raison, 2016; Straub, 2017). SCI can also impair normal immune function, leading to increased vulnerability to infections, poor response to vaccines, and/or cancer (Furman et al., 2019; Shen-Orr et al., 2016).

**Figure 2:**
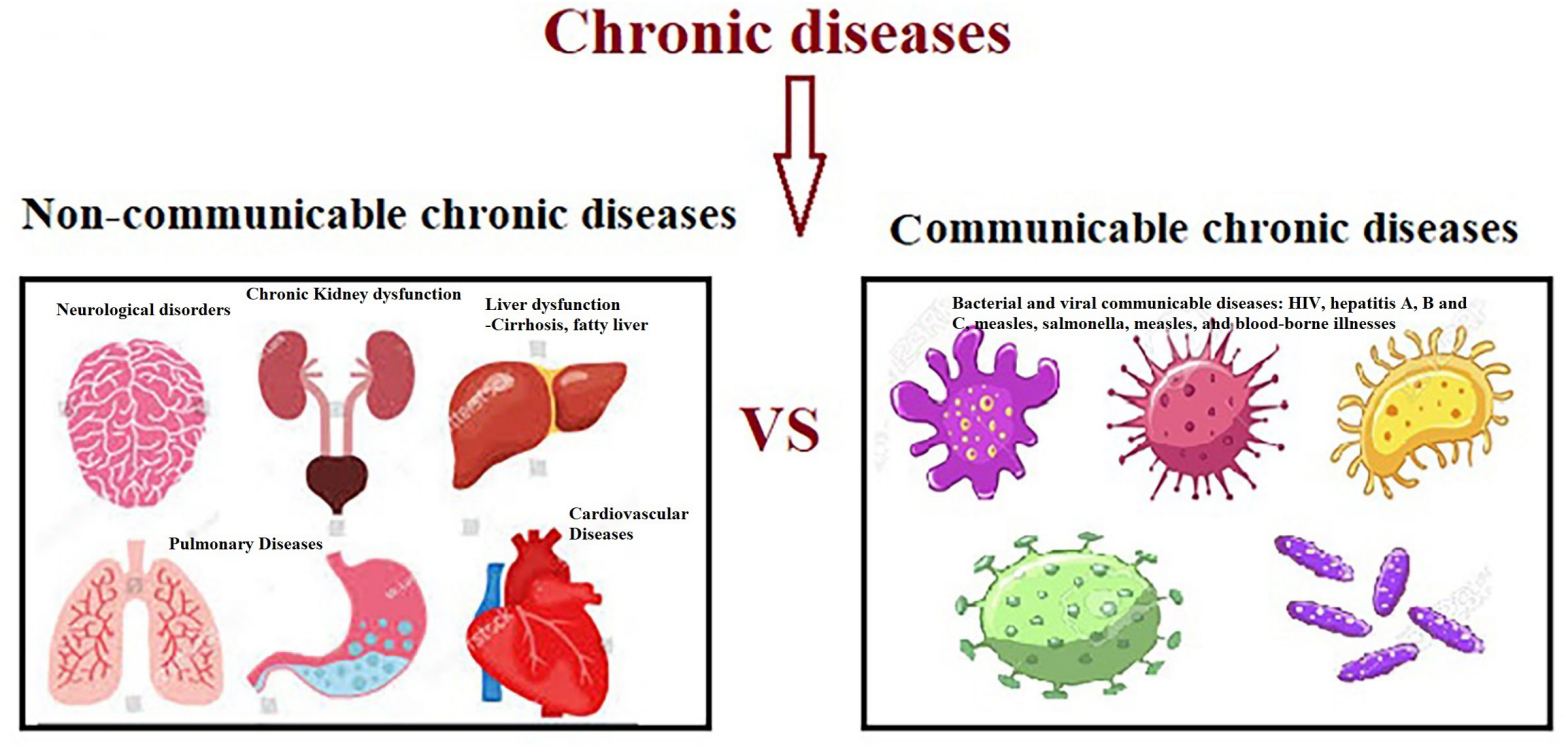
Non-communicable and communicable of chronic diseases.

Clinically, SCI-related impairment can be severe including high risk of developing metabolic syndrome, a triad of hyperglycemia, hypertension, and dyslipidemia (Hotamisligil, 2017), cardiovascular disease (CVD) and chronic kidney disease (Miller & Raison, 2016), various types of cancer (Taniguchi & Karin, 2018), depression (Miller & Raison, 2016), neurodegenerative and autoimmune diseases (Heneka, Kummer & Latz, 2014), and osteoporosis (Redlich & Smolen, 2012) (Fig. 2).

In fact, a meta-analysis study including over one hundred and sixty thousand subjects distributed among fifty four long-term prospective studies, concluded that elevated C-reactive protein (CRP) levels, a circulating SCI indicator, were linked to a higher risk for developing CVD disease, stroke, and even mortality (Y. Li et al., 2017). Interestingly, the most plausible evidence suggesting a relationship between SCI and risk of disease has emerged from randomized controlled trials (RCTs) that have looked at drugs or biologics that aims at modifying certain pro-inflammatory cytokines, such as interleukin (IL)-1 *β* and tumor necrosis factor (TNF)- *α*. A remarkable evidence emerged regarding the improvement in inulin sensitivity associated the use of TNF- *α* inhibitor to manage rheumatoid arthritis, this was demonstrated in a meta-analysis of eight RCTs that involved two hundred and sixty subjects (Burska, Sakthiswary & Sattar, 2015). In addition, patients who received TNF- *α* inhibitor to manage rheumatoid arthritis had also lower likelihood of becoming affected by Alzheimer’s disease (Chou et al., 2016).

Therefore, autoantibodies have been proposed as biomarkers for early-stage detection of cancers. Recently, Wang et al. (178) suggested that serum TAAbs can improve early detection of colorectal cancer through the application of serological proteome analysis (SERPA) to initially screen total proteins from cancer tissues to identify autoantibodies discrepancies between healthy individuals and cancer patients. Consequently, protein microarrays and enzyme linked immunosorbent assays (ELISAs) were used to further monitor and ascertain their value in diagnosing cancer patients. They measured the predictive value of either a single autoantibodies or panels of multiple autoantibodies using receiver operating characteristics (ROCs) to identify valuable biomarkers of early-stage colon cancer (Wang et al., 2020).

#### Mitochondrial dysfunction and chronic diseases

Mitochondria are cellular organelles that convert energetic substrates such as lipids, glucose, and oxygen into energy. These organelles have a double membrane, circular DNA molecules, and the ability to interact with each other (Braschi & McBride, 2010). Constant mitochondrion-mitochondrion interaction takes place through dynamic processes of membrane fusion and fission. These interactions alter mitochondrial morphology and simultaneously modulate the organelles’ function (Westermann, 2010).

Mitochondrial dysfunction, characterized by loss of the electron transport chain efficiency and reduction in the ability to generate adenosine triphosphate (ATP), is a common character of all chronic diseases (Green, Galluzzi & Kroemer, 2011; Swerdlow, 2011).

Mitochondrial dysfunction, chronic inflammation, and oxidative stress are involved in most NCDs such as cancer, atherosclerosis, Parkinson’s disease, Alzheimer’s disease, other neurodegenerative diseases, heart and lung disturbances, diabetes, obesity, and autoimmune diseases (Ij, 2017). In addition to the impairment of mitochondrial function, obesity and obesity-related complications are universally associated with NCDs.

**Figure 3:**
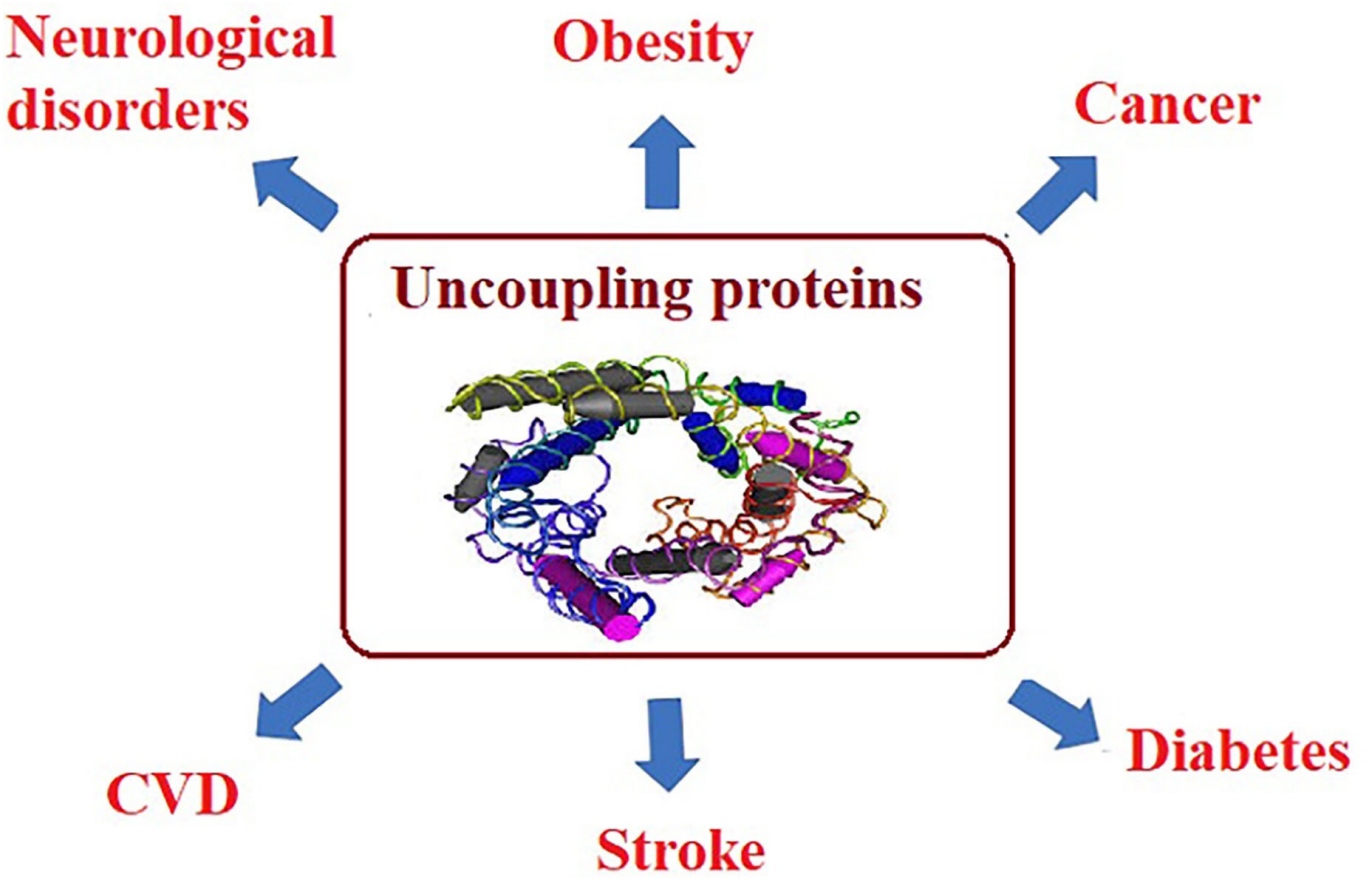
Uncoupling proteins as mitochondrial dysfunction-related etiological mechanisms of most chronic diseases.

For example, in muscle tissues, mitochondrial dysfunction leads to the reduction of fatty acid oxidation and the inhibition of glucose transport, indicating that insulin-stimulated glucose transport is reduced as a hallmark of insulin resistance and type 2 diabetes. Mitochondrial ROS have also been correlated to the amplified activity of uncoupling proteins (UCP), which uncouples ATP synthesis from electron transport. UCP activity leads to heat generation without ATP production. Long-term reductions in ATP levels, affect multiple cellular signaling mechanisms associated with chronic disease (Brondani et al., 2012; Fedorenko, Lishko & Kirichok, 2012). Usually, NCDs, such as cancer, obesity, and diabetes are accompanied with high ROS levels which serve as markers for oxidative stress and elevated concentrations of free fatty acids (FFA) (Matsuda & Shimomura, 2013). Since ROS and FFA are important molecular signals that activate UCP2, it is essential to understand and clarify the relationship between UCP2 and chronic diseases. UCP is a family of mitochondrial anion proteins existing in the inner mitochondrial membrane. Since their discovery, they have attracted significant attention due to their contribution to the mitochondrial-mediated oxidative stress and impaired energy metabolism. Therefore, UCP2 is an important target to consider in the efforts attempting to control the prevalence of multiple chronic diseases (Sreedhar & Zhao, 2017) as presented in Fig. 3.

It is generally accepted nowadays, that mitochondrial dysfunction is involved in the etiological mechanisms of several chronic diseases, including cardiac, liver and kidney disorders, and neurodegenerative diseases (*e.g.*, Parkinson’s disease and Alzheimer’s disease) (Geto et al., 2020).

**Table 1:** Summary of the most important biomarkers of common NCDs.

NCDs	Biomarker	Pathways	Refernces
Diabetes	**HbA1c** HbA1c has moderate sensitivity in diagnosing diabetes, increased HbA1c levels are associated with increased morbidity and mortality	HbA1c is a reflection of chronic glycemia	Sherwani et al. (2016)
	**Adiponectin:** Lower levels of adiponectin are associated with increased IR and obesity.	IR, increased oxidative stress, and lipid oxidation may cause chronic shifts in glutathione synthesis leading to elevated α-HB levels	Ziemke & Mantzoros (2010)
	Fetuin-A (FetA)	Correlates with increased risk of developing T2DM and associated complications.	Dorcely et al. (2017)
	α-HB organic acid byproduct	T2DM	Gall et al. (2010)
CVD	**C-reactive protein (CRP)**	Elevated in Inflammatory conditions	Badimon et al. (2018)
	**Cardiac troponin I (cTnI)**	Acute myocardial infarction and acute coronary syndrome	Apple, Collinson & IFCC Task Force on Clinical Applications of Cardiac Biomarkers (2012) Welsh et al. (2019)
	**D-dimer**	Thrombosis, ischemic heart disease, acute aortic dissection, cardiovascular mortality	Chen et al. (2017) Tokita et al. (2009)
	**Tetranectin**	Elevated as marker of Presence and severity of diseased coronary arteries	Chen et al. (2015)
	Secreted frizzled-related proteins sFRPs	Early stages of MI and function as Wnt signaling antagonists	Huang & Huang (2020)
	Serum amyloid A	Acute phase protein that increases the expression of pro-thrombotic and pro-inflammatory molecules	Brea et al. (2009) Shridas & Tannock (2019) Krishack et al. (2015)
Cancer	Serum Fascin autoantibodies	Esophageal squamous cell carcinoma	Shimada et al. (2003) Xu et al. (2014) Li et al. (2017a) Li et al. (2017b) Peng et al. (2016) Chen et al. (2017) Xu et al. (2017) Ortiz et al. (2010) Shang et al. (2018)
	Levels of tumor-associated autoantibodies (AAbs) panel of seven TAAs (p53, PGP9.5, SOX2, GAGE7, GBU4-5, CAGE and MAGEA1) combined with CT scan.	Lung cancer	Li et al. (2017a) Li et al. (2017b)
	carcino- embryonic antigen (CEA Carcinogen embryonic antigen	Colon cancer	Chan et al. (2010) Li et al. (2013)
	Alpha feto-protein CA-125	Liver cancer Ovarian cancer	Li et al. (2018) McGuire et al. (2019)
	CA-19-9	Gastrointestinal cancer	Duffy et al. (2010)
COPD	Bronchoalveolar Angiogenic growth factor overexpression	Overexpression of VEGF and PIGF	Kuhlmann et al. (2013)
	Sputum and serum Calprotectin	Track changes in lung inflammation during an exacerbation of cystic fibrosis	Gray et al. (2010)
	Serum C-reactive protein(CRP)	Elevated in acute exacerbation of COPD	Ruiz-González et al. (2016)
	H_2_O_2_ in exhaled breath	Measurement of oxidative stress in	Guatura et al. (2000)
	F2-isoprostanes	pulmonary diseases	Van de Kant et al. (2012)
	Nitric oxide (NO) in exhaled breath	Inflammatory lung disorders, e.g., asthma and Rhinosinusitis	Taylor et al. (2006)

### Microorganisms and chronic disease

Numerous chronic diseases are not a direct result of microbial infection; however, certain microbial infections are associated with chronic disease such as Chlamydia infection and CVD (Joshi et al., 2013). The relationship between microbial infection and chronic disease is believed to be somewhat complicated. For example, early stages of infection can lead to lifelong chronic illness (Joshi et al., 2013), in addition, microbial infections can, indirectly, predispose an individual to chronic disease as suggested in the case of maternal infection prompting neurodevelopmental disorders such as autism (O’Connor, Taylor & Hughes, 2006). The identification of infection as a trigger for chronic disease is critically important because it may permit earlier laboratory diagnosis and treatment of the disease and provide significant prevention prospects. Relations between communicable transferable agents and cancer of the liver, cervix, skin, and gastrointestinal tract have suggested that more cancers may have an infectious origin (Ewald & Swain Ewald, 2015). Human papillomavirus (HPV), hepatitis C (HCV), hepatitis B (HBV) viruses, and helicobacter pylori accounted for high rate of human malignancies (Boshoff & Weiss, 2002).

*Chlamydophila pneumoniae* (CP) can cause lung infections such as pneumonia (Grayston et al., 1993). It has been reported that individuals with IgG antibody to CP were at higher risk of developing myocardial infarction (MI) suggesting a role for CP infection in CVD (Bjerke, 2011). Additionally, live CP and CP-specific T-cells have been detected in coronary and carotid atherosclerotic plaque (Mosorin et al., 2000; Yamashita et al., 1998) but not in adjacent healthy un-infected tissues (Kuo et al., 1993; Maass et al., 1998). Table 1 demonstrates the most important biomarkers of common NCDs

Periodontitis is caused by microorganisms that stick to and grow on tooth surfaces, provoking violent immune responses. Periodontitis has been associated with increased inflammation at different sites of the body, as shown by increased levels of CRP and IL-6 (Anderson & Muhlestein, 2005; Joshi et al., 2021). Periodontitis is usually concomitant with an elevated risk of MI, stroke, and atherosclerosis (Beck et al., 2019; Pussinen et al., 2011). For example, *Porphyromonas gingivalis* (*P. gingivalis*) seropositivity was associated with an increased risk of stroke during a 15-year follow-up period; compared with seronegative subjects (Pussinen et al., 2007). Interestingly, high *P. gingivalis* IgA antibody titer can predict MI independently of classic cardiovascular risk factors (Beck et al., 2019; Pussinen et al., 2011).

The possible role of microbial agents such as CP, herpes simplex-1 virus (HSV-1) and *P. gingivalis* in the development of Alzheimer’s disease have been studied. CP is capable of sticking to tissues and crossing the blood–brain barrier (BBB), activating endothelial cells, and finally causing inflammation and pathophysiology associated with the clinical presentation of Alzheimer’s disease (Itzhaki et al., 2004; Letenneur et al., 2008). Balin et al. (Balin et al., 1998) reported that 90% of postmortem brain biopsies from Alzheimer’s disease patients were positive for CP.HSV-1 antibodies have been associated with Alzheimer’s diagnosis (Letenneur et al., 2008).

Current evidence suggests that some chronic inflammatory diseases are mediated or affected by the dysfunction of the gut microbiota and its metabolic end-products (Tilg et al., 2020). However, probiotics as a healthy beneficial bacterium may not have the same positive effect on all subjects or on all chronic diseases (Durack & Lynch, 2019).

There is evidence to suggest that microbial variety is reduced considerably in CD Gut microbiome diversity of CD patients compared to healthy patients but are comparable in pattern across all patients with each disease (Blaser, 2017; Felizardo et al., 2016). Compared with controls, CD patients have an overgrowth of *E. faecium* and several Proteobacteria (Mondot, Boudinot & Lantz, 2016). It is well documented that alterations of the microbiota could contribute to obesity (Ridaura et al., 2013). Decreased abundance of Bacteroidetes in obese individuals usually ensues as a result of caloric restriction and weight loss (Manichanh et al., 2006). In an attempt to find biomarkers related to altered microbiome in CD, a recent study has highlighted the role of gut microbiota in the production of the pro-atherosclerotic metabolite known as trimethylamine N-oxide (TMAO) following ingestion of lecithin (in eggs). An elevated plasma TMAO level was associated with increased risk of a major adverse cardiovascular event independent of other risk factors (Tang & Hazen, 2017).

Interestingly, patients with chronic kidney diseases (CKD) can be differentiated form healthy individuals by analyzing the microbiota of the gut. The CKD group had a significantly greater richness of *Bacteroidetes* and *Proteobacteria* and lower richness of *Firmicutes* compared with the group comprised of healthy subjects (Lun et al., 2019). Furthermore, *Holdemanella*,* Megamonas*,* Prevotella* 2, *Dielma*, and *Scardovia*, could serve as markers of the advancement of CKD and hemodialysis need [145]. It has been found that *p*-cresyl sulfate and indoxyl sulfate, which are bacterial metabolites, can be used as markers of CKD severity. Both metabolites, prior to their removal by tubular secretion, are known to bind with and are quickly released from circulating albumin (Leong & Sirich, 2016; Meijers et al., 2008). Decreased removal of *p*-cresyl and indoxyl sulfates by the kidneys is believed to be the reason for their associated increased levels with CKD advancement (Wu et al., 2019).

### Recent advances in biomarkers of major NCDs

Biological markers are defined as “a characteristic that is objectively measured and evaluated as an indicator of normal biological processes, pathogenic processes, or pharmacologic responses to a therapeutic intervention”. This broad definition encompasses a wide range of factors that are associated with disease processes and termination thereof. Existing research on diabetes, which is a growing global epidemic, is aiming on understanding how the weakened physiological processes and clinical presentation of diabetes are related and how they can be used for the recording of novel predictive biomarkers that could accordingly improve diagnostic, controlling, and treatment strategies (Xie, Chan & Ma, 2018).

The current practice in the management of diabetes is generally based on the measurement of fasting glucose and HbA1c (Xie, Chan & Ma, 2018). Interestingly, the pathophysiology of both pre-diabetic and diabetic cases is insulin resistance in peripheral tissue, which initiates a compensatory hyper-synthesis of insulin by the beta cells of the pancreas (Bornfeldt & Tabas, 2011; Groop, Forsblom & Thomas, 2005; Varvel et al., 2014). Later-stage of diabetes is characterized by pancreatic beta cell dysfunction leading to relative or absolute hypoinsulinemia. Based on this etiology, Varvel et al. (2014) were able to use a comprehensive panel of 19 blood- biomarkers and glycaemia related measures in addition to insulin resistance and beta cell function markers. It was found that the whole panel (*i.e.,* 19 biomarkers) was effective in the early identification of insulin resistance which could help improve glycemic management in a high proportion of patients. No individual, or even small subset of these markers were responsible for the remarkable increase of the sensitivity. Therefore, the high cost associated with using this 19-biomarker panel preclude its use as a practical routine clinical investigation.

Borges et al. (2011) used another panel of biomarkers to characterize protein heterogeneity in diabetic patients and to test the possibility to categorize T2DM, and CVD comorbidities. Using this panel was effective in categorizing patients into five disease subgroups: T2DM, T2DM with congestive heart failure (CHF), T2DM/CHF and previous myocardial infarction, non-diabetes with CHF/MI, and non-diabetes with CHF. They utilized principal component analysis (PCA) to evaluate markers that have a shared pathological pathway in an attempt to discover the least number of biomarkers that are providing the maximum degree of discrimination between diabetic subgroups. As such, oxidation markers were compressed to sulfoxidised apolipoprotein A-1, and apolipoprotein C-I (apoCI), glycation markers to albumin, b2-microglobulin, cystatin C, vitamin D binding protein, and C-reactive protein; and truncation markers to apoCI, and N-terminal di-peptide truncations. Through the use of PCA it was again possible to reduce the panel of biomarkers into a single metric for each of the three pathological mechanisms (glycation, oxidation and truncation) which were then tested for its predictive power using receiver operating characteristic (ROC) and area under the curve AUCs. Remarkably, it was found that that biomarkers of protein oxidation and truncation, but not glycation, as displayed by ROCs, are able to discriminate between subjects with and without complicated diabetes. However, Borges et al. (2011) were unable to differentiate, in their study, between DM subgroups and healthy controls. Looking at available evidence collectively, it seems reasonable to conclude that protein modification-based biomarkers are not promising for the identification of individuals with T2DM, however, grouping of patients based on their comorbidities could be a useful application for protein modification-based biomarkers.

**Figure 4:**
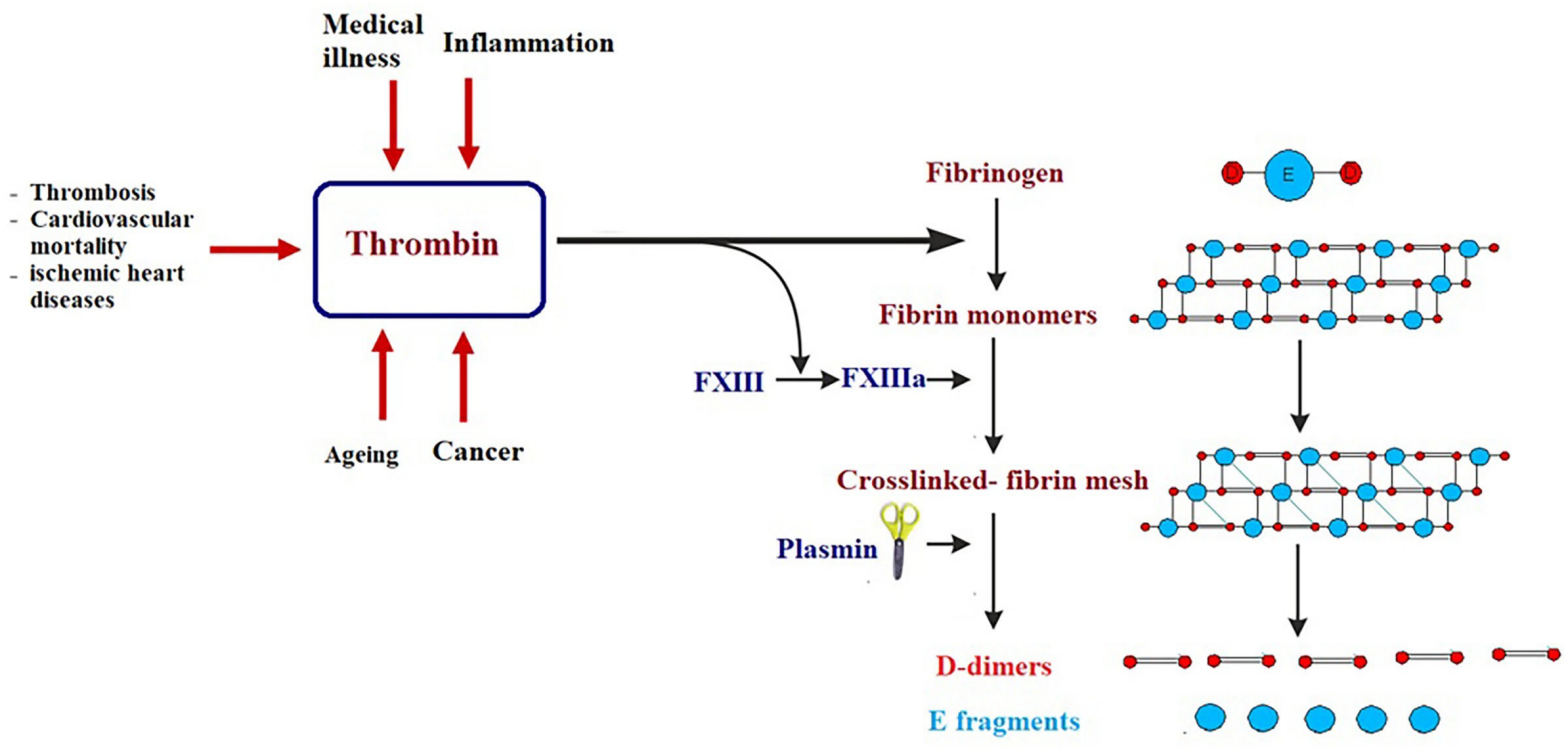
Simplified illustration of D-dimer generation as late-stage biomarker of cardiovascular diseases. Thrombin transforms fibrinogen to fibrin monomers that are composed of a central E-domain and 2 outer D-domains. Fibrin monomers polymerize, thus making an unstable fibrin mesh. Activated blood clotting factor XIII cross-links the D-domains, which supports the fibrin network. Fibrin-bound plasmin degrades the fibrin mesh into soluble fragments: D-dimers and E fragments.

Certain biomarkers could be risk factors themselves and therefore probable targets of treatment (Jiang et al., 2015; Townsend et al., 2018). It is well known that obesity, hypertension, smoking, gender, age, LDL cholesterol, diabetes, and inactive lifestyle are risk factors for CVD, nevertheless, these factors can only be used to categorize patients at high risk but never to avoid or predict an acute or fatal attack, such as myocardial infarction (MI). Framingham Risk Score, which is an algorithm that calculates a 10-year risk of developing cardiovascular acute attack, usually uses these risk factors (Wilson et al., 1998). Low risk individuals have a score of less than 10%, while intermediate risk is shown at 10–20%, and high risk is seen when the score is over 20% (Wilson et al., 1998). There are currently several clinical biomarkers that are associated with cardiovascular attack. These biomarkers include: C-reactive protein (CRP), cardiac troponins I and T (cTnI and cTnT), B-type natriuretic peptides (BNP and NT-proBNP), and D-dimer (Chen & Burnett, 2017; Omland & White, 2017). CRP levels predict cardiovascular morbidity and high levels of CRP are directly associated with future cardiovascular threats (Pfützner & Forst, 2006; Strandberg & Tilvis, 2000). The cardiac troponins cTnI and cTnT are significant biomarkers in diagnosing acute MI and in stratifying hazards of acute coronary illness (Apple, Collinson & IFCC Task Force on Clinical Applications of Cardiac Biomarkers, 2012; Scirica & Morrow, 2004). The B-type natriuretic peptides (BNP and NT-proBNP) are considered diagnostic biomarkers of heart failure (Chen et al., 2014; Di Angelantonio et al., 2009). D-dimer is a biomarker of thrombosis, cardiovascular mortality, acute aortic dissection, and ischemic heart disease (Alehagen, Dahlström & Lindahl, 2004; Tokita et al., 2009). Although these biomarkers are normally used in clinical diagnosis and have enabled doctors to save lives, they usually discover cardiovascular disease after an attack has already happened (late-stage biomarkers) (Fig. 4). The challenge is to find biomarkers that detect early-stage CVD in order to significantly reduce morbidity and mortality associated with cardiovascular attacks and improve prognosis.

In an attempt to find biomarkers for early-stage CVD, protein profiling using proteomic techniques and mRNA screening utilizing microarray platform and RNA sequencing was of great help to identify abnormal levels of proteins and differential expression of genes in early stages of CVD (167). With these biomarkers, etiological mechanisms of CVDs can be better understood, and the prediction of CVDs can be enhanced. Ceholski et al. (2012) reported that phospholamban PLN-R9C can serve as an early-stage biomarker of cardiomyopathy and later heart failure. Another early detection biomarker is myeloperoxidase (MPO), which is an enzyme that catalyzes the formation of hyperchlorite from chloride and hydrogen peroxide and is usually elevated under inflammatory conditions (Heslop, Frohlich & Hill, 2010; Hochholzer, Morrow & Giugliano, 2010). MPO breaks down the collagen layer in atherosclerotic plaque, thus leading to its destruction. High MPO levels are considered an early detection biomarker of CVD due to their correlation with atheroma instability. Clinical trials have found that elevated MPO levels are early indicators of coronary artery disease (Teng et al., 2017), even before detection by angiography or cardiac troponin levels (169). Secreted frizzled-related proteins (sFRPs) are produced at early stages of MI and function as Wnt signaling antagonists (Huang & Huang, 2020). When the Wnt pathway is antagonized by sFRP3, a pro-apoptotic pathway typical of MI and heart failure is activated (Huang & Huang, 2020). Thus, MI, heart failure, and its adverse outcomes are associated with high circulating levels of sFRP3, suggesting a potential role for sFRP3 as an early-stage diagnostic biomarker of heart failure (Askevold et al., 2014). Although there are more CVD associated biomarkers in the literature, the discovery of a logarithmic panel of biomarkers is still necessary to improve the prognostic accuracy of CVDs.

Cancer is one of the chief causes of death in the developed world with 9.6 million cancer related deaths in 2018 (Ferlay et al., 2018). Despite this large number of deaths, there is a remarkable reduction in the mortality rate of cancer patients. This reduction in mortality is attributed to improvements in treatment, changing patterns in cancer risk lifestyle factors, and screening for early-stage biomarkers (Siegel et al., 2015). Lately, autoantibodies were assessed as potential biomarkers for cancer diagnosis because their appearance may be months or years prior to the clinical confirmation of cancer diagnosis.

Li et al. (2005) demonstrated that anti-p53, which is a tumor suppressor protein autoantibody, was significantly associated with a consequent growth of malignancy with a predictive value of 0.76, with 3.5 years as an average time before diagnosis. Tumor-associated autoantibodies (TAAbs) have been found during the conversion to malignancy (Poletaev et al., 2015). Autoantibodies can be prompted by immune cascades leading to their presence at steadily high levels even with low levels of the corresponding antigen (Poletaev et al., 2015; Wang et al., 2019).

### Advances in the applications of multivariate biomarkers of NCDs

Recent years have witnessed a rapidly increasing interest in the multivariate approach both in biomarkers research and all biomedical sciences. A PubMed search for scientific articles using the keywords “biomarker” and “multivariate” found 1,139 articles from January 1, 1950 to December 31, 2010 (61 years). In contrast, searching the same keywords between January 1, 2011 and March 13, 2021 (10 years, 2 months, and 13 days) returned 10,319 articles, 1,750 of which were in the past year alone [accessed March 13, 2021]. A mounting interest is anticipated to continue into foreseeable future given the many advantages of multivariate analysis and the growing richness in experimental data obtained using newly introduced technologies, such as high-throughput sequencing and single-cell multiomics.

Univariate biomarkers are often sufficient for clinical applications involving the diagnosis and management of disease conditions that rely on one or a few individual markers. For example, monitoring blood glucose or glycated hemoglobin (HbA1c) is sufficient for the diagnosis and management of diabetes mellitus (Kerner, Brückel & German Diabetes Association, 2014). Similarly, serum levels of aminotransferases [alanine aminotransferase (ALT) and aspartate aminotransferase (AST)], gamma glutamyl transferase (GGT), alkaline phosphatase (ALP), bilirubin, and other surrogate biomarkers related to hepatocellular and cholestatic injury have been instrumental in the diagnosis and management of liver disease (Agrawal, Dhiman & Limdi, 2016). In both examples, two biomarkers (glucose and HbA1c) or several biomarkers (ALT, AST, ALP, GGT, bilirubin, and others) have been used in a univariate manner, without the use of a multivariate algorithm. Early detection and outcome prediction of more complex disease conditions, however, are not possible to effectively accomplish using a univariate approach. Even in the case of liver function biomarkers that have been used in clinical practice for decades, the univariate approach had its own limitations. These limitations led to the advent of combined scoring systems aimed to enhance the clinical utility of the biomarkers, such as Child–Pugh–Turcotte (CTP) and Model for End Stage Liver Disease (MELD) scores (Agrawal, Dhiman & Limdi, 2016). The use and limitations of liver function biomarkers is elegantly reviewed elsewhere (Agrawal, Dhiman & Limdi, 2016). Furthermore, the complications associated with biomarker discovery are paramount with even more mechanistically or clinically complex conditions, such as autism spectrum disorder (ASD), where the need for a multivariate approach is indeed inexorable. Many biomarkers have been suggested for ASD, but a widely accepted consensus has not been reached up until the date of this writing. Even though an efficacious biomarker based on routinely testable laboratory variables has yet to enter medical practice, preliminary studies have already demonstrated the superiority of multivariate biomarkers and their improved accuracy and specificity compared to univariate biomarkers (Abruzzo et al., 2015; Hassan et al., 2018). Alzheimer’s disease (AD), with its complex, multifaceted pathogenesis (Tiwari et al., 2019), is another illustration of the superiority of the multivariate approach in biomarker discovery. Low levels of amyloid beta 42 (A *β*42) and elevated total and phosphorylated tau (t-tau and p-tau, respectively) in the cerebrospinal fluid (CSF) have been used for the early diagnosis of AD and progression from mild cognitive impairment (MCI) to AD (Llano et al., 2017). However, more accurate prediction of AD and MCI-to-AD conversion was achieved using a multivariate profile based on CSF proteomics in a recent study (Guan et al., 2017). A recent study examined 21 plasma metals in search for biomarkers that maximize the discrimination of AD patients from healthy controls. The two groups were completely separated in the discriminant scores plot and manganese, aluminum, lithium, and copper were identified as the most important distinguishing variables between the groups (Badea et al., 2019). Similarly, a multivariate biomarkers profile based on magnetic resonance imaging parameters outperformed a univariate approach using the same parameters in a mouse model of AD (Zetterberg & Burnham, 2019). The superiority of the multivariate approach in accurately identifying AD patients and MCI patients at high risk of progression to AD has been reviewed by Zetterberg & Burnham (2019).

**Figure 5:**
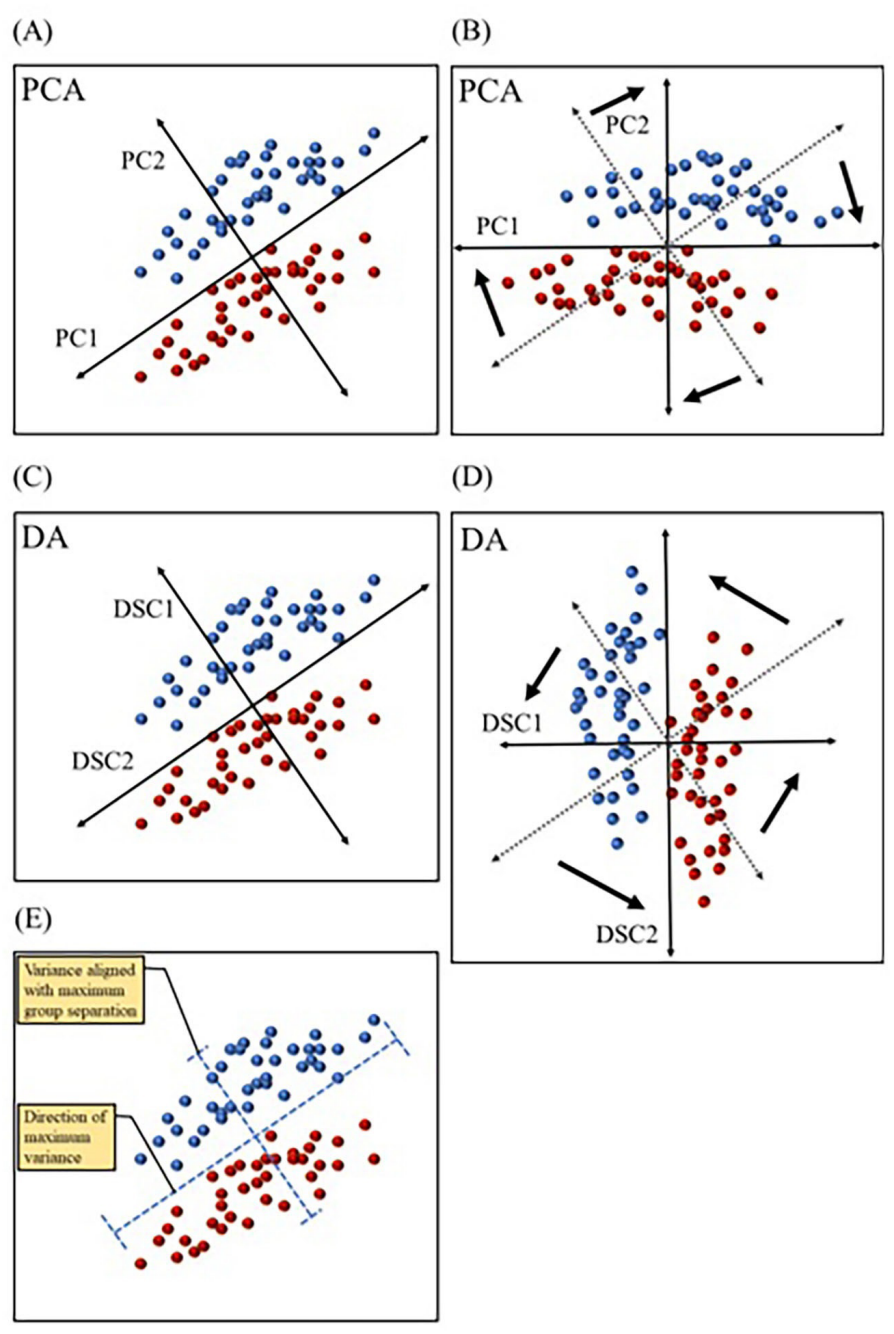
Comparing principal component analysis and discriminant analysis. Principal component analysis (PCA) and discriminant analysis (DA) both combine correlated variables into common vectors (A–D) known as principal components (PCs) and discriminant functions (DSCs) in PCA and DA, respectively. The first component (PC1) in principal component analysis is designed to account for the most variance, while ignoring predefined groups (A & B). The first discriminant function (DSC1) in discriminant analysis is designed to maximize separation between predefined group (C & D). PCs and DSCs can be rotated and used to plot the data in a new coordinate system (B & D). Data variation (variance) aligned with PC1 and DSC1 are illustrated (E). Dotted axes (B & D) represent the original directions of PCs and DSCs before rotation.

Multiple multivariate methods have been used in biomarker discovery. Principal component analysis (PCA) is a dimension reduction technique that simplifies the interpretation of multivariate data. PCA consolidates correlated variables into “artificial variables” or vectors, known as principal components (PCs). The goal of PCA is to maximize the amount of variance explained by each of the PCs. Therefore, it computes the first principal component (PC1) in such a way so that it is aligned with the direction of most dispersion among datapoints (*i.e.,* the direction of most variance) (Warner, 2007). Figure 5 depicts fictitious bivariate data graphed with each of the two variables represented by the x or y axis. In this dataset, individual datapoints are divided into two groups, but the direction of the widest variation or scatter of datapoints is not the same direction that separates groups (Fig. 5E). PC1 in the PCA graph (Fig. 5A) is aligned in the direction covering the most variance, with no regard to group separation. This technique is useful when the goal is to examine natural partitioning of data based on a range of variables. PCA is also useful in identifying outliers. Another technique that also combines correlated variables into common vectors is discriminant analysis (DA). DA differs from PCA in that the former maximizes group separation (Robotti, 2013). In other words, DA picks the vectors, or discriminant functions (DSCs) as they are called in DA, that best distinguish between user defined groups, with the first discriminant function (DSC1) being the most discriminatory (Fig. 5C). This method is advantageous in studies that aim to identify the efficacy of a range of variables in distinguishing between groups, such as in the case of biomarkers of various disease conditions. Both PCs and DSCs can be used to plot the data based on combined scores that represent various contributions from the original variables (Figs. 5B & 5D). Another commonly used technique is logistic regression (LR), which is also designed to separate groups but is computationally distinct from DA. LR relies on the probability of belonging to a group (Robotti, 2013). For example, if data is collected from a specific patient population and age-matched healthy controls, LR may be used to calculate the probability of belonging to the patient group for each participant. This allows the classification of participants into patient and control groups based on multivariate data. More detailed description of these and other multivariate techniques has been reviewed by Robotti and colleagues (Robotti, 2013).

## Conclusions

It is important to prospectively assess biomarkers in a variety of large populations over a prolonged duration, and to confirm their relationship to the severity of chronic disease, prior to the development of complications and/or mortality when considering their use in clinical practice. Although advances in proteomics technologies, sample conditioning, and analysis methods have greatly improved productivity and efficiency in biomarker discovery, biomarker verification and validation remains a significant, costly, and high-risk undertaking in the commercial development and deployment of novel biomarkers for some chronic diseases.

## List of Abbreviations

AD: Alzheimer’s disease
AGEs: Advanced glycation end products
AIDS: Acquired immunodeficiency syndrome
ALP: Alkaline phosphatase
ALT: Alanine aminotransferase
apoCI: apolipoprotein C-I
ASD: Autism spectrum disorder
AST: Aspartate aminotransferase
ATP: Adenosine triphosphate
A β42: Amyloid beta 42
BBB: blood–brain barrier
BNP: B-type natriuretic peptides
CAP: Community acquired pneumonia
CDs: Communicable diseases
CHF: Congestive heart failure
CKD: Chronic kidney disease
CP: Chlamydophila pneumonia
CRP: C-reactive protein
CSF: Cerebrospinal fluid
cTnI and cTnT: Cardiac troponins I and T
CTP: Child–Pugh–Turcotte
CVD: Cardiovascular disease
DA: Discriminant analysis
DSCs: Discriminant functions
DSC1: First discriminant function
ELISAs: Enzyme linked immunosorbent assays
ESR: Erythrocyte sedimentation rate
FFA: Free fatty acids
GCF: Gingival crevicular fluid
GGT: Gamma glutamyl transferase
GSH-Px: Glutathione peroxidase
HbA1c: Glycated hemoglobin
HBV: Hepatitis B
HCV: Hepatitis C
HD: Huntington’s disease
HPV: Human papillomavirus
HSV-1: Herpes simplex-1 virus
IL: Interleukin
LR: Logistic regression
MELD: Model for End Stage Liver Disease
MI: Myocardial infarction
MPO: Myeloperoxidase
NADPH: Nicotinamide adenine dinucleotide phosphate
NCDs: non-communicable diseases
NDs: Neurological disorders
NFK-B: Nuclear factor kappa-B
NO: Nitric oxide
PCA: Principal component analysis
PD: Parkinson’s disease
*P. gingivalis*: *Porphyromonas gingivalis*
*PKC*: Protein kinase C
PMNs: Polymorphonuclear leukocytes
QOL: Quality of life
RAGEs: Advanced glycation end receptors
RCTs: Randomized controlled trials
ROCs: Receiver operating characteristics
RONS: Reactive oxygen and nitrogen species
ROS: Reactive oxygen species
RON: Reactive nitrogen species
SERPA: Serological proteome analysis
SCI: Systemic chronic inflammation
SOD: Superoxide dismutase
TAAbs: Tumor associated autoantibodies
T2D: Type 2 diabetes
TMAO: Trimethylamine N-oxide
TNF: Tumor necrosis factor
UCP: Uncoupling proteins
WBC: White blood cell
WHO: World Health Organization

## Additional Information and Declarations

### Data Availability

The following information was supplied regarding data availability: This article is a literature review and we did not have raw data.
